# Supplementary material for: Decoupling of respiration rates and abundance in marine prokaryoplankton
Source: Nature. 2022 Dec 7;612(7941):764–70. doi: 10.1038/s41586-022-05505-3 (PMC9771814; doi:10.1038/s41586-022-05505-3)
Supplement: Supplementary file 2 — Reporting Summary [file 41586_2022_5505_MOESM2_ESM.pdf]

nature portfolio

Corresponding author(s): Ramunas Stepanauskas

Last updated by author(s): 03/10/2022

Reporting Summary

Nature Portfolio wishes to improve the reproducibility of the work that we publish. This form provides structure for consistency and transparency in reporting. For further information on Nature Portfolio policies, see our Editorial Policies and the Editorial Policy Checklist.

Statistics

For all statistical analyses, confirm that the following items are present in the figure legend, table legend, main text, or Methods section.

n/a	Confirmed	
☐	☒	The exact sample size (*n*) for each experimental group/condition, given as a discrete number and unit of measurement
☐	☒	A statement on whether measurements were taken from distinct samples or whether the same sample was measured repeatedly
☐	☒	The statistical test(s) used AND whether they are one- or two-sided<br>*Only common tests should be described solely by name; describe more complex techniques in the Methods section.*
☐	☒	A description of all covariates tested
☐	☒	A description of any assumptions or corrections, such as tests of normality and adjustment for multiple comparisons
☐	☒	A full description of the statistical parameters including central tendency (e.g. means) or other basic estimates (e.g. regression coefficient) AND variation (e.g. standard deviation) or associated estimates of uncertainty (e.g. confidence intervals)
☐	☒	For null hypothesis testing, the test statistic (e.g. *F*, *t*, *r*) with confidence intervals, effect sizes, degrees of freedom and *P* value noted<br>*Give P values as exact values whenever suitable.*
☒	☐	For Bayesian analysis, information on the choice of priors and Markov chain Monte Carlo settings
☒	☐	For hierarchical and complex designs, identification of the appropriate level for tests and full reporting of outcomes
☒	☐	Estimates of effect sizes (e.g. Cohen's *d*, Pearson's *r*), indicating how they were calculated

Our web collection on statistics for biologists contains articles on many of the points above.

Software and code

Policy information about availability of computer code

Data collection	Data was generated using the commercially available programs FlowJo (BD; for Flow cytometry analyses), and NextSeq (Illumina; for DNA sequencing).
Data analysis	Data analysis was performed using multiple commercial and open source software (described in materials and methods) and custom scripts to process outputs. Custom code is available upon request.

For manuscripts utilizing custom algorithms or software that are central to the research but not yet described in published literature, software must be made available to editors and reviewers. We strongly encourage code deposition in a community repository (e.g. GitHub). See the Nature Portfolio guidelines for submitting code & software for further information.

Data

Policy information about availability of data

All manuscripts must include a data availability statement. This statement should provide the following information, where applicable:

- Accession codes, unique identifiers, or web links for publicly available datasets
- A description of any restrictions on data availability
- For clinical datasets or third party data, please ensure that the statement adheres to our policy

All sequence data has been deposited at NCBI (BioProject PRJNA846736) and the Open Science Framework (https://osf.io/r2un6)

Field-specific reporting

Please select the one below that is the best fit for your research. If you are not sure, read the appropriate sections before making your selection.

☐ Life sciences ☐ Behavioural & social sciences ☒ Ecological, evolutionary & environmental sciences

For a reference copy of the document with all sections, see nature.com/documents/nr-reporting-summary-flat.pdf

Ecological, evolutionary & environmental sciences study design

All studies must disclose on these points even when the disclosure is negative.

Study description	This manuscript reports results from multiple experiments with diverse study designs. Detailed information has been provided in Supplemental Materials.
Research sample	This manuscript reports results from a broad spectrum of marine microorganisms. Detailed information has been provided in Supplemental Materials.
Sampling strategy	This manuscript reports results from multiple experiments with diverse study designs. Detailed information has been provided in Supplemental Materials.
Data collection	This manuscript reports results from multiple experiments with diverse study designs. Detailed information has been provided in Supplemental Materials.
Timing and spatial scale	This manuscript reports results from multiple experiments with diverse study designs. Detailed information has been provided in Supplemental Materials.
Data exclusions	This manuscript reports results from multiple experiments with diverse study designs. Detailed information has been provided in Supplemental Materials.
Reproducibility	All experiments were repeated when possible.
Randomization	This manuscript reports results from multiple experiments with diverse study designs. Detailed information has been provided in Supplemental Materials.
Blinding	Blinding was not possible, due to the unique nature of each analyzed sample.

Did the study involve field work? ☒ Yes ☐ No

Field work, collection and transport

Field conditions	Detailed information has been provided in Supplemental Materials.
Location	Detailed information has been provided in Supplemental Materials.
Access & import/export	Seawater samples from the Gulf of Maine were collected off the dock of the Bigelow Laboratory in East Boothbay, Maine, US, and did not require permit. Seawater samples from AT42-11 were collected with consent of the Government of Canada.
Disturbance	This study did not cause any environmental disturbance.

Reporting for specific materials, systems and methods

We require information from authors about some types of materials, experimental systems and methods used in many studies. Here, indicate whether each material, system or method listed is relevant to your study. If you are not sure if a list item applies to your research, read the appropriate section before selecting a response.

Materials & experimental systems

n/a	Involved in the study	
☒	☐	Antibodies
☒	☐	Eukaryotic cell lines
☒	☐	Palaeontology and archaeology
☒	☐	Animals and other organisms
☒	☐	Human research participants
☒	☐	Clinical data
☒	☐	Dual use research of concern

Methods

n/a	Involved in the study	
☒	☐	ChIP-seq
☐	☒	Flow cytometry
☒	☐	MRI-based neuroimaging

Flow Cytometry

Plots

Confirm that:

☒ The axis labels state the marker and fluorochrome used (e.g. CD4-FITC).

☒ The axis scales are clearly visible. Include numbers along axes only for bottom left plot of group (a 'group' is an analysis of identical markers).

☐ All plots are contour plots with outliers or pseudocolor plots.

☐ A numerical value for number of cells or percentage (with statistics) is provided.

Methodology

Sample preparation	Samples of seawater and bacterial cultures were labeled using either SYTO-9 or RedoxSensor Green (Thermo Fisher Scientific)
Instrument	Influx Mariner (BD) and ZE5 (Bio-Rad)
Software	Sortware and FlowJo (BD)
Cell population abundance	An individual cell was collected in each microplate well. The accuracy of droplet deposition into microplate wells was confirmed several times during each sort day by sorting 3.46 µm diameter SPHERO Rainbow Fluorescent Particles (Spherotech, Inc., Lake Forest, IL) and microscopically examining their presence at the bottom of each well. In these examinations, <2% wells did not contain beads and <0.4% wells contained more than one bead.
Gating strategy	Cells were gated based on particle green fluorescence (531/40 BP), forward scatter, and the ratio of green versus red fluorescence (692/40 BP - for improved discrimination of cells from detrital particles). Killed negative controls were used to determine RSG background fluorescence.

☒ Tick this box to confirm that a figure exemplifying the gating strategy is provided in the Supplementary Information.
